# On the Seasonal Occurrence and Abundance of the Zika Virus Vector Mosquito Aedes Aegypti in the Contiguous United States

**DOI:** 10.1371/currents.outbreaks.50dfc7f46798675fc63e7d7da563da76

**Published:** 2016-03-16

**Authors:** Andrew J. Monaghan, Cory W. Morin, Daniel F. Steinhoff, Olga Wilhelmi, Mary Hayden, Dale A. Quattrochi, Michael Reiskind, Alun L. Lloyd, Kirk Smith, Chris A. Schmidt, Paige E. Scalf, Kacey Ernst

## Abstract

Introduction: An ongoing Zika virus pandemic in Latin America and the Caribbean has raised concerns that travel-related introduction of Zika virus could initiate local transmission in the United States (U.S.) by its primary vector, the mosquito Aedes aegypti.

Methods: We employed meteorologically driven models for 2006-2015 to simulate the potential seasonal abundance of adult Aedes aegypti for fifty cities within or near the margins of its known U.S. range. Mosquito abundance results were analyzed alongside travel and socioeconomic factors that are proxies of viral introduction and vulnerability to human-vector contact.

Results: Meteorological conditions are largely unsuitable for Aedes aegypti over the U.S. during winter months (December-March), except in southern Florida and south Texas where comparatively warm conditions can sustain low-to-moderate potential mosquito abundance. Meteorological conditions are suitable for Aedes aegypti across all fifty cities during peak summer months (July-September), though the mosquito has not been documented in all cities. Simulations indicate the highest mosquito abundance occurs in the Southeast and south Texas where locally acquired cases of Aedes-transmitted viruses have been reported previously. Cities in southern Florida and south Texas are at the nexus of high seasonal suitability for Aedes aegypti and strong potential for travel-related virus introduction. Higher poverty rates in cities along the U.S.-Mexico border may correlate with factors that increase human exposure to Aedes aegypti.

Discussion: Our results can inform baseline risk for local Zika virus transmission in the U.S. and the optimal timing of vector control activities, and underscore the need for enhanced surveillance for Aedes mosquitoes and Aedes-transmitted viruses.

## Introduction

Zika virus (ZIKAV), a flavivirus, was first isolated from a primate in 1947 in the Zika forest of Uganda and in 1948 from *Aedes (Ae.) africanus* mosquitos in the same location^1^. Serological evidence of ZIKAV transmission in humans has been reported since 1951, but until recently, transmission was confined to ecological niches in the equatorial regions of Africa and Asia^2^. In 2007, an outbreak of ZIKAV in Yap State, Federated States of Micronesia, was the first documented epidemic outside Africa or Asia^3^^,^ 4. Over 28,000 cases were reported in 2013-2014 in French Polynesia, and nearby island nations followed^5^^,^ 6. Introduced into Brazil in 2015, ZIKAV has spread explosively across Latin America and the Caribbean, and is now pandemic, with over 20 countries reporting autochthonous transmission^7^^,^ 8 ^,^ 9. This follows the introduction and subsequent pandemic of chikungunya virus (CHIKV) in the Western Hemisphere in late 2013^10^^,^ 11. *Aedes* mosquitoes transmit ZIKAV and CHIKV, as well as dengue (DENV) and yellow fever (YFV) viruses. Over the past several decades the range of *Aedes* mosquitoes has expanded across the Americas^12^^,^ 13. Although *Ae. albopictus* is thought to be a competent vector of ZIKAV^14^, *Ae. aegypti* has been implicated as the primary transmitter of the virus in human populations in the ongoing outbreak in the Americas^15^.

ZIKAV infections are commonly characterized by fever, maculopapular rash, arthralgia or conjunctivitis. Clinical illness is usually mild, occurs in approximately 20% of people infected with ZIKAV, and symptoms typically last for a week^8^. However, in mid-November 2015, the Pan American Health Organization issued an epidemiological alert^16^ based on an unusual increase in the number of children born with microcephaly in northeast Brazil that coincided with the arrival of ZIKAV^17^. Although no causal links have been established, in the aftermath of a previous outbreak of ZIKAV in French Polynesia during 2014-2015, a notable increase in central nervous system malformations in newborns and infants was also registered^18^. Additionally, in both Brazil and French Polynesia, following recent illnesses with ZIKAV-like symptoms, authorities detected patients with neurological syndromes such as Guillain-Barré^18^.

As of 22 February 2016, all cases of ZIKAV reported in the contiguous United States were associated with travel or sexual transmission^72^. Given the potential link between exposure to ZIKAV during pregnancy and microcephaly, the U.S. Centers for Disease Control and Prevention (CDC) has recommended pregnant women consider postponing non-essential travel and urges all travelers to take enhanced precautions in areas where ZIKAV is circulating^19^. The risk for local transmission of ZIKAV has been discussed widely in the media and scientific and public health communities. Sporadic outbreaks of DENV and CHIKV have already occurred in the United States and there is concern that local outbreaks of ZIKAV could follow^20^^,^ 21 ^,^ 22. Prior DENV outbreak investigations in the U.S.-Mexico border region suggest improved socioeconomic conditions in the United States may minimize vector-human contact and reduce the risk of *Aedes*-transmitted arboviruses^20^^,^ 23 ^,^ 24. Aside from sexual transmission that has been reported in comparatively few instances^25^, for local transmission to occur ZIKAV would have to be introduced by infected travelers or infected mosquitoes and become sustained in local *Aedes* mosquito populations. While *Ae. aegypti* and *Ae. albopictus* are established across much of the southern United States (Fig. 1), mosquito populations in many areas are only present seasonally. This seasonality varies according to local meteorological conditions, and thus can provide one measure of potential for ZIKAV transmission (i.e., local transmission may not be sustained if competent vector mosquitoes are not present). In addition, assuming transmission dynamics are similar to DENV, risk of ZIKAV introduction can be modulated by population travel patterns, human exposure to *Aedes* mosquitoes, and vector control response to travel-associated cases^26^^,^ 27. To better understand the risk for local ZIKAV transmission in the United States, we simulated the potential abundance of adult *Ae. aegypti* mosquitoes across fifty cities using meteorologically-driven life cycle models, and identified proxies of travel-related introduction and of human exposure to vectors to provide context for the model results.

**Figure:**
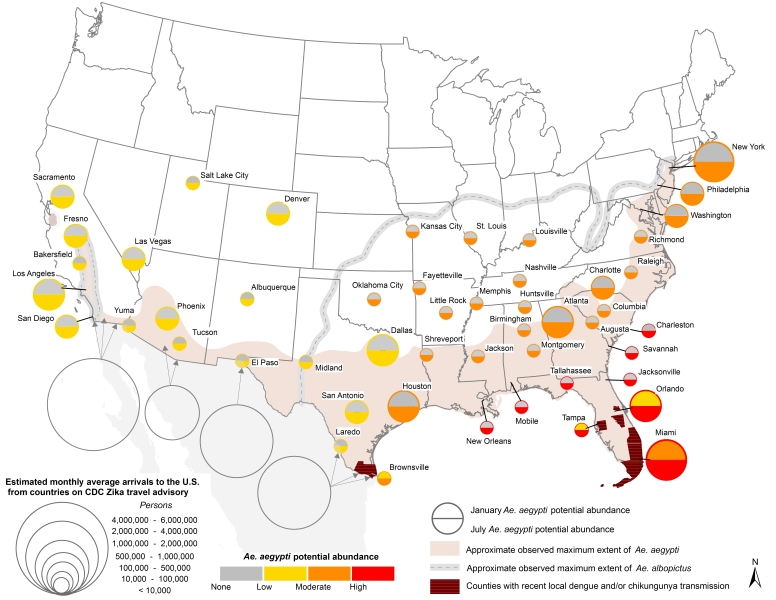
**Fig. 1.** U.S. map showing 1) *Ae. aegypti* potential abundance for Jan/July (colored circles), 2) approximate maximum known range of *Ae. aegypti* (shaded regions) and *Ae. albopictus* (gray dashed lines), and 3) monthly average number arrivals to the U.S. by air and land from countries on the CDC Zika travel advisory. Additional details can be found in the text.

## Data and Methods

Cities

We chose fifty U.S. cities within and near the known range of *Ae. aegypti* (Fig. 1, Table 1). These cities were selected because they may be meteorologically suitable for *Ae. aegypti* for at least part of the year, though not all have recorded the presence of *Ae. aegypti* to date. In general, seasonal and annual temperatures within the study cities decrease from south-to-north, and precipitation decreases from east-to-west. These gradients are modified in the vicinity of the Appalachian and Rocky Mountains, and by proximity to the ocean.

**Figure:**
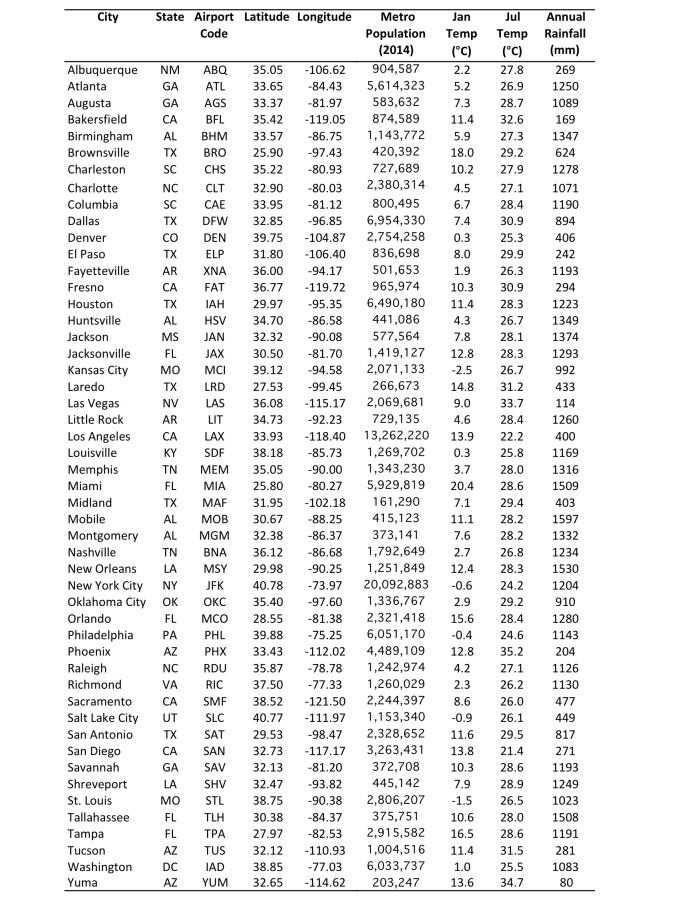
**Table 1.** Coordinates, population^28^ and climate characteristics^30^^,^ 31 for the 50 study cities.

*Ae. aegypti* life cycle modeling

To estimate the seasonal abundance of adult *Ae. aegypti* we employed two life cycle models, Skeeter Buster^32^ and DyMSiM^33^. Both models simulate the egg, larval, pupal and adult life stages as a function of daily meteorological inputs and user specified information about container habitats. Using two models enabled the quantification and reduction of model uncertainty; previous studies have shown that model ensembles provide a more accurate result than those from individual models^34^^,^ 35 ^,^ 36. Both models simulated daily counts of adult *Ae. aegypti* mosquitoes per 1 m^2^ of water surface for a multi-year period, as described below. The amount of water in containers was modulated primarily by rainfall and evaporation, but a fraction of water representing about 5-10% of the maximum due to rainfall was made available in containers year-round to account for the possibility that water may be available even during periods of little or no rainfall due to humans filling containers intentionally (e.g., via birdbaths, kiddie pools, etc.) or unintentionally (e.g., during lawn irrigation). Human-filled containers can represent an important source of water for *Ae. aegypti* habitat in arid and semi-arid regions^73^. For both models, releasing new cohorts of eggs each month prevented extinction of mosquitoes, or (in the case of DyMSiM) when the population of any life stage reduced to zero. Releases ranged from 80-100 eggs per container. This choice essentially assumes that *Aedes* eggs are frequently reintroduced into areas through transportation networks^37^ or that eggs are able to survive through periods when weather is unfavorable for development. There is strong evidence that *Ae. aegypti* eggs are capable of surviving even in harsh winter conditions as a result of being laid in favorable cryptic environments such as subterranean habitats^38^.

The DyMSiM and Skeeter Buster models differ primarily in their treatments of containers and stochasticity. DyMSiM assumes one large water-filled container per city^33^; in this study a surface area of 1 m^2^ was specified for each DyMSiM container, and water volume fluctuated as a function of rainfall and evaporation but did not drop below 0.001 m^3^ or rise above 0.02 m^3^. Skeeter Buster operates at the level of individual containers, and therefore was configured to simulate 10 adjacent households per city, each having numerous containers with a surface area totaling 1 m^2^. Each container had a surface area of 0.01 m^2^ and could be filled to a maximum depth of 0.2 m. As with DyMSiM, Skeeter Buster water depth was allowed to vary as a function of rainfall and evaporation, and manual filling was performed randomly for 5% of containers on average every 7 days. The resulting simulated *Ae. aegypti* adult counts were then averaged over the 10 homes. In this manner, the initial adult mosquito abundance results for both DyMSiM and Skeeter Buster were expressed for each 1 m^2^ of water surface area per city; these values were then normalized for presentation, as described below. Sheltering of containers (via shading) was also considered for both models because *Ae. aegypti* prefers shaded environments^29^. DyMSiM treated sheltering by reducing evaporation among 50% of containers and limiting the maximum water temperature to <34^o^C. Skeeter Buster assumed a sun exposure of 0.1 (i.e., 90% shade) for containers. Additional descriptions of both models can be found in the publications cited above, and model inputs and other study-specific details are described below.

Simulations were conducted with both models for the most recent eleven years, 2005-2015. The meteorological fields needed to drive the models – daily precipitation, humidity and maximum and minimum near-surface temperature – were derived from the 1/8^th^ degree hourly meteorological forcing dataset for the North American Land Data Assimilation Phase 2 (NLDAS-2)^30^^,^ 31. The widely used NLDAS-2 forcing data have been employed for numerous health-related applications over North America requiring high fidelity meteorological data^39^^,^ 40. The NLDAS-2 data were bilinearly interpolated to the coordinates of each city from the four nearest grid points.

Once the simulations were complete, the results for year 2005 were discarded to allow for model burn-in. Then, for each city (n=50) and each day of the year (n=365) the mean adult *Ae. aegypti* abundance was computed from the remaining 10-year sample (2006-2015) for each model. Ensemble mean values were computed by taking the geometric mean of the daily mosquito abundance values from both models. Using a geometric mean rather than an arithmetic mean allows the two models to be weighted equally despite having different mean *Ae. aegypti* abundance values. Next, the average daily abundance was computed for Miami for May-October (Julian days 121-304) for both models and the ensemble mean, and then the mean daily abundance for each city and day of year was expressed as a percentage of this average. In this manner, the potential abundance patterns across cities and for each day were normalized with respect to the average warm season conditions for Miami. Finally, these normalized daily values were averaged for each month and city for both models and the ensemble mean. We chose Miami and the May-October period because it is the warmest and wettest time of year in south Florida, a time when *Ae. aegypti* is known to be abundant^41^ and when most transmission of *Ae. aegypti*-borne viruses has occurred in the past^42^. May-October in Miami thus represents high environmental suitability for *Ae. aegypti*.

The normalized daily values of adult *Ae. aegypti* potential abundance were placed into four categories to facilitate comparison across space and time: 1) “None to low” for normalized abundances <2% the value of the May-Oct Miami mean; 2) “Low to moderate” (2-25%); 3) “Moderate to high” (25-75%); and 4) “High” (>75%). These cut points were chosen to provide a proxy of the lower quartile, the interquartile range, and the upper quartile with respect to Miami values. The “None to low” cut-point of 2% rules out extremely low abundance values that are unlikely to be sufficient to generate substantial risk for human contact.

Results of simulations of adult *Ae. aegypti* populations are referred to in terms of potential abundance in order to emphasize that they indicate meteorological suitability for the mosquito but not necessarily that it is present or will occur in a given location. For example, we find that Denver, CO has low-to-moderate potential abundance for the mosquito during the summer months, however as of this writing presence of the mosquito has not been detected there. Possible reasons that *Ae. aegypti* does not occur in some cities that exhibit meteorological suitability during part of the year include a lack of eggs being introduced during the warm months, or a lack of water availability (particularly in western U.S. cities).

Model validation data

Validating the simulations of potential seasonal abundance of *Ae. aegypti* was challenging because there are few observational records of seasonally-resolved *Ae. aegypti* abundance (for any life stage) that can provide comparative benchmarks. Focks et al.^43^ present graphical records of weekly ovitrap accumulations of *Ae. aegypti* eggs for 1981-1985 from a number of southeastern U.S. cities, based on unpublished data from CDC. We were unable to obtain the ovitrap data from Focks et al.^43^ at the time this manuscript was submitted, which limited us to a qualitative comparison of our simulated adult abundance to the ovitrap data. However, we did find quantitative seasonal records for two cities, in two different climatic settings and compared the seasonality of observed vs. modeled *Ae. aegypti* abundance. The first dataset contains *Ae. aegypti* immature abundance collected from 30 sites covering a 675 km^2^ area of Palm Beach, FL, collected over 27 four-week periods from 2006-2008^41^. *Aedes aegypti* immature abundance was determined from samples collected in the field from ovitraps (eggs) or from water in containers (larvae), and reared in a laboratory to fourth-instar larvae or pupal stages for identification to species. A comparison of results from a variety of adult and egg trapping methods for *Ae. aegypti* 44 showed that the seasonal cycle of abundance for trapped adults versus trapped eggs was similar, suggesting that validating our normalized simulated adult abundance results to normalized egg/larval abundance results^41^ is a reasonable approach. Palm Beach is located just north of Miami, FL and has similar climatic conditions; therefore we consider this dataset to be representative of Miami. The second dataset contains unpublished monthly adult abundance for 750 trapping sites across Phoenix, AZ collected over 10 years from 2006-2015 by co-author KAS and colleagues. Samples were collected with CDC light traps baited with CO2 and laboratory-identified to species. Miami has a warm humid climate, and Phoenix has a warm desert climate. Both cities have distinct wet and dry seasons.

Transportation data

To understand the potential for human introduction of ZIKAV, we determined the number of air travelers arriving in the U.S. from airports in Latin American countries and territories listed on the CDC Zika travel advisory as of 29 January 2016^45^. These countries and territories included the U.S. Virgin Islands, Dominican Republic, Commonwealth of Puerto Rico, Barbados, Bolivia, Brazil, Colombia, Ecuador, El Salvador, French Guiana, Guadeloupe, Guatemala, Guyana, Haiti, Honduras, Martinique, Mexico, Panama, Paraguay, Saint Martin, Suriname, and Venezuela. We did not include Cape Verde and Samoa, two countries not in Latin America that account for a very small fraction of passengers into the United States. Monthly estimated passenger counts on outbound international flights to airports in these countries originating from the U.S. were obtained for year 2014 from a database maintained by the U.S. Department of Transportation^46^. Inbound passenger counts were not available, however the data curator confirmed that they are approximately equal to outbound counts (personal communication, Michael Lane, USDOT); we confirmed this by analyzing international flight data for domestic carriers only (for which both inbound and outbound counts exist) and found that inbound and outbound passenger counts differ by <1% for monthly and longer timescales^47^. Passenger data were only available for the international leg of each flight and therefore do not indicate the number of passengers traveling on to secondary and tertiary U.S. destinations. Therefore, it is likely that passenger numbers are overestimated for large international hubs (e.g., Miami, Atlanta, New York, Los Angeles) and underestimated for smaller airports that primarily serve domestic passengers (e.g., New Orleans, Nashville, Jacksonville). Additionally, it is probable that some passengers were from Latin American countries not on the CDC ZIKAV travel advisory (e.g., Argentina, Chile) who had traveled through major international airports in countries on the advisory.

Because Mexico shares a border with the U.S. and is on the CDC ZIKAV travel advisory, we also obtained year 2014 estimates of the monthly number of persons crossing into the U.S. from Mexico along the land border by passenger vehicle, bus, train or as pedestrians^48^.

We did not obtain data for cruise ship arrivals, though a number of ports along the Gulf of Mexico and the eastern seaboard depart to areas of the Caribbean with ongoing Zika transmission^49^.

DENV and CHIKV transmission data

To provide information about the location of *Aedes*-transmitted virus outbreaks we mapped counties with confirmed local DENV and CHIKV transmission in the contiguous U.S. from 1 January 2010–6 February 2016. Data were obtained from The Florida Department of Health^42^ and from the CDC ArboNET via the United States Geological Survey Disease Mapper^50^. It is noteworthy that DENV transmission occurred in the contiguous U.S. prior to 2010, however those reported cases were more difficult to confirm and additionally were mainly from counties in Florida and Texas already identified as having DENV transmission using the (more reliable) data from 2010 onward.

Human exposure data

Vector-human contact is likely modulated by socioeconomic factors. Impoverished communities are at higher risk of introduction of infectious diseases^51^. Poverty has been linked to a number of indicators of elevated exposure to mosquitoes such as lower usage rates of air conditioning and less efficient cooling options^52^, poorer housing infrastructure such as screening of windows^53^, as well as decreased access to safe water and sanitation^54^. To better understand potential human exposure to adult *Ae. aegypti*, we obtained and mapped 2014 county-level data for the percentage of households living below the poverty line from the U.S. Census Bureau^55^. The poverty line varies according to the size of a family and ages of its members. Thresholds do not vary geographically but they are updated annually for inflation. In 2014, the poverty line was (for example) defined by an annual income of $12,316 for a household with a single adult under 65 years, and $24,008 for a household with two adults and two children under 18 years.

Maximum *Aedes* geographic range data

A recent compendium of observed *Ae. aegypti* and *Ae. albopictus* presence locations collected between 1960-2014^56^^,^ 57 ^,^ 58 was used to provide background information on the maximum known geographic range of both mosquitoes in the U.S. (Fig. S1). These data were augmented by surveillance data of both *Aedes* species across southern and central California^59^. Known ranges for both species were then established by outlining areas in which presence locations were recorded (Fig. 1). Such information is useful for placing our results for simulated seasonal potential abundance of *Ae. aegypti* into context, as the mosquito may not exist in some cities where seasonally suitable meteorological conditions exist, for example because eggs are unable to overwinter^60^. Additionally, mapping known occurrences of *Aedes* mosquitoes enables comparison of the simulated potential range of *Ae. aegypti* to the known range of *Ae. albopictus*, which is also a competent vector of ZIKAV in some regions^14^. This is important because *Ae. albopictus* (which was not simulated in this study) has greater cold tolerance than *Ae. aegypti*, and therefore could facilitate seasonal ZIKAV transmission risk in more northerly U.S. cities where *Ae. aegypti* is not found^61^.

## Results

Simulated potential abundance of *Ae. aegypti* adults is zero or near-zero throughout the U.S. in January except in southern Florida and Texas (Fig. 1). By mid-July, all fifty cities are meteorologically suitable for *Ae. aegypti*, with far southeastern cities being suitable for high abundance, and other eastern cities being suitable for moderate-to-high abundance. Cities in the western U.S. are suitable for low-to-moderate abundance of *Ae. aegypti*. Some cities that are suitable in mid-summer are not within the present known range for *Ae. aegypti* (e.g., Denver, Albuquerque, Louisville). As the season progresses from winter to summer *Ae. aegypti* potential abundance begins to increase in April in the southeastern U.S. and some cities in Arizona (Fig. 2). By June nearly all cities exhibit the potential for at least low-to-moderate abundance, and most eastern cities are suitable for moderate-to-high abundance. Conditions are most suitable in July, August and September during the warmest (and in many cities wettest) time of year. Conditions in the southern and western states remain suitable through November, and by December are largely unsuitable again, except for southern Florida and Texas.

**Figure:**
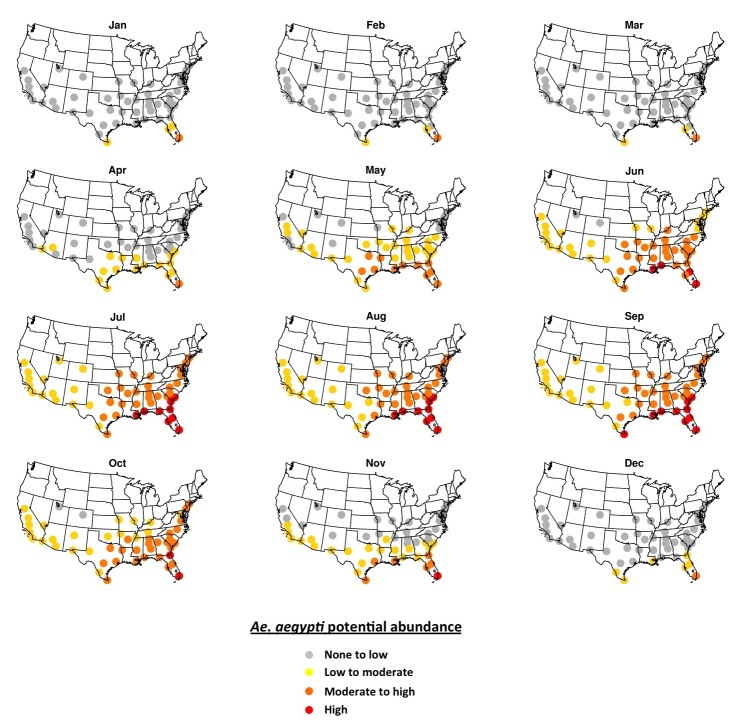
**Fig. 2.**The 2006-2015 ensemble mean monthly average *Ae. aegypti* potential abundance.

Model results demonstrated a similar seasonal cycle compared to the observed *Ae. aegypti* abundance at our two validation sites (Fig. 3). However, in Miami the observed onset and cessation of peak summer abundance in June-July occurs more rapidly than in the simulations, which have a broader peak that extends later into the summer. Interestingly, our climate data indicate that daily maximum and minimum temperatures and rainfall in August-September are similar to June-July conditions, suggesting that very high abundance might otherwise be expected to extend into these later summer months. The difference between peak and off-season abundance in Miami is also more pronounced in the observations. A qualitative comparison of simulated adult abundance to observed average weekly ovitrap accumulations in Tampa^43^ (not shown) similarly indicates a more pronounced difference between peak and off-season abundance than is simulated. However, for Southeast cities with somewhat cooler winters like Jacksonville and New Orleans the seasonal cycle of simulated adult *Ae. aegypti* abundance is similar to the observed ovitrap accumulations. It is possible that the broader, lower amplitude seasonality of *Ae. aegypti* abundance that is simulated in the warmer Southeast cities like Miami and Tampa occurs because the models do not account for interspecies competition between *Ae. aegypti* and *Ae. albopictus*, which may favor *Ae. albopictus* in areas with longer warm and wet periods^41^. Or, it is also possible that monthly egg introductions specified in the model simulations may lead to broader peaks in adult *Ae. aegypti* abundance (i.e., more eggs may be available in the models compared to reality). In Phoenix the observed peak abundance in August, during the monsoon season, is resolved by the simulations, as is the substantial difference between peak and off-season abundance (off-season abundance is basically zero in both the observations and simulations). An early-season peak in the simulations during April-May, which is not observed, is due to the small amount of water maintained in the simulated containers during dry periods to accommodate the potential for manual filling of containers. In reality manually filled containers may be relatively uncommon in Phoenix, though allowing for a modest amount of human-mediated water availability in simulations during the dry season can be useful for determining when conditions are warm enough for *Ae. aegypti* development and survival. **** ****Additionally, the models may underestimate the effects of low humidity (i.e., desiccation) during the dry season on adult survival, which may be particularly important in arid environments^62^. Vector control activities likely also lead to differences between observed and simulated *Ae. aegypti* populations in both cities (the simulations do not account for mosquito control). Given the uncertainty in the observations and the limitations of the models, the objective of the comparison in Fig. 3 is to evaluate whether the models can simulate the general seasonal cycle including higher abundances during observed peak months, and lower abundances during observed off-peak months, for these two cities that represent different climatic settings; we conclude that the model simulations meet this basic objective.

**Figure:**
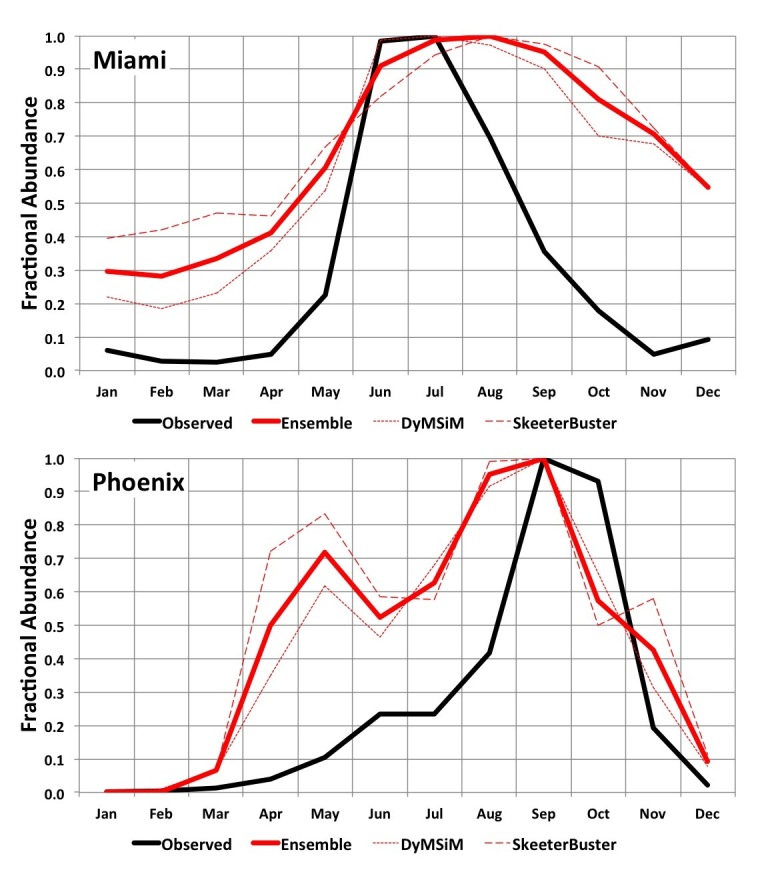
**Fig. 3.** 2006-2015 average simulated monthly *Ae. aegypti* potential abundance versus average observed abundance for Miami (2006-2008; top) and Phoenix (2006-2015; bottom). Results are expressed as a fraction of the maximum monthly abundance for each dataset in order to facilitate comparison (otherwise abundance measures will vary widely due to how simulated versus observed mosquitoes are quantified and the areal extents they represent).

The estimated monthly average number of persons arriving by air from countries on the CDC Zika virus travel advisory – which provides a proxy for the potential for virus introduction – is greatest in large cities with major international airports (Fig. 1). Cities having both high potential *Ae. aegypti* abundance and large volumes of air passengers include Miami, Houston and Orlando. It is also noteworthy that nearly five times as many persons cross the U.S.-Mexico border per month than arrive by air in all fifty cities combined.

Travel patterns fluctuate seasonally (Fig. 4). Importantly, July and August are the two months with the highest estimated number of passengers arriving by air from countries on the CDC Zika travel advisory. These months fall within the season of highest meteorological suitability for *Ae. aegypti* across the fifty cities. These months also have comparatively high traffic across the U.S.-Mexico border. The holiday months of December and January also have relatively high estimated numbers of persons arriving both by air and across the U.S.-Mexico border; these are months having no-to-low *Ae. aegypti* potential abundance in most cities, although there may be low-to-moderate potential abundance in cities like Brownsville, Miami and Orlando. Although rare in winter, one case of locally-acquired DENV was reported in January 2011 in Miami-Dade county^42^, indicating that in some years off-season *Aedes* populations may be abundant enough in these cities to enable virus transmission.

**Figure:**
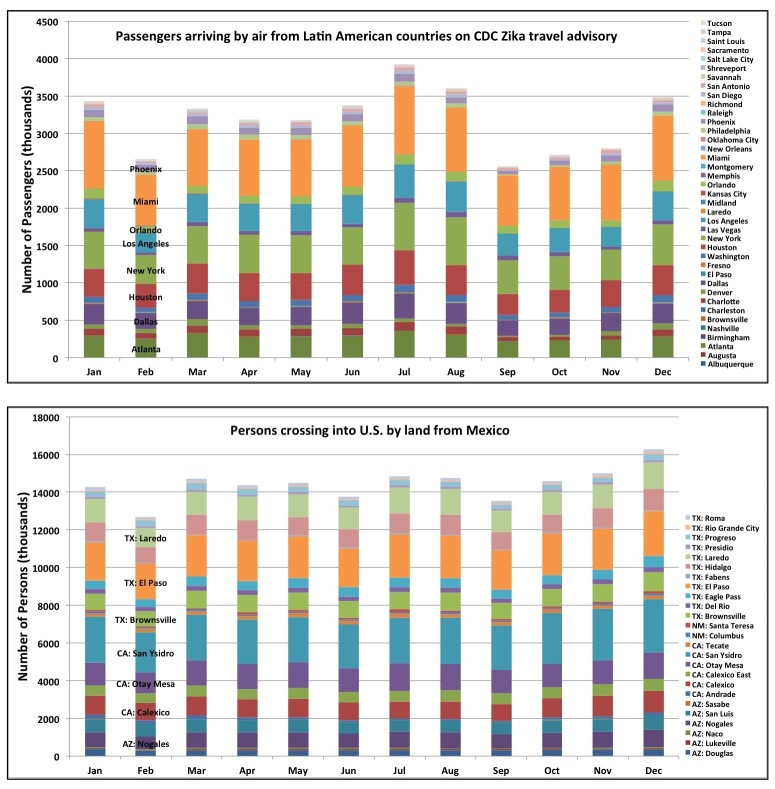
**Fig. 4.** 2014 monthly number of persons arriving by air from Latin American countries on the CDC Zika travel advisory (top), and by land from Mexico (bottom). Sources for data can be found in the text.

High poverty rates, an indicator of potential vector-human contact, are particularly prevalent in the southern U.S. where seasonal abundance of *Ae. aegypti* is simulated to be highest (Fig. 5). Many of the poorest counties across the geographic area under study are rural and unlikely to be inhabited by *Ae. aegypti* given its proclivity for urban areas^55^. *Ae. albopictus*, however, is present across the rural South (Fig. S1) and may be a competent vector of Zika virus^14^. The poorest urban areas include the counties that encompass Laredo, TX and Brownsville, TX, where poverty rates are >30%, as well as Yuma, AZ and Miami, FL (>20%). A number of other southern cities in this study are situated within counties having poverty rates > 15%.

**Figure:**
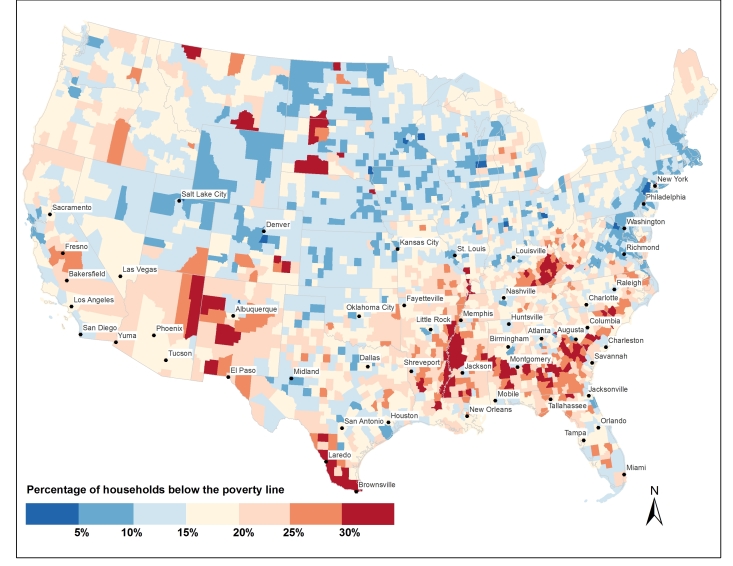
**Fig. 5.** 2014 percentage of households below the poverty line by U.S. county. Source of data can be found in text.

## Discussion

Numerous studies have produced global maps of general climatic suitability for *Ae. aegypti* 56 ^,^ 63 ^,^ 64. Our results for peak warm season *Ae. aegypti* potential abundance over the U.S. compare reasonably with the maps published in these previous studies. Fewer studies have explored seasonal suitability for *Ae. aegypti*. Brady et al.^60^ mapped weekly temperature-based suitability for *Ae. aegypti* and *Ae. albopictus* oviposition globally (they also examined weekly temperature suitability for introduction and persistence of DENV transmission for both mosquitoes). Here, we employ temperature, precipitation and humidity fields to drive two process-based life cycle models and simulate the daily potential abundance of *Ae. aegypti* for the most recent ten years at fifty cities across the U.S. While these simulations are subject to limitations as discussed below, they are an important step toward improved understanding of the spatial and seasonal variability of *Ae. aegypti* in the U.S. and the periods of higher risk of ZIKAV introduction.

We find that meteorological conditions are largely unsuitable for *Ae. aegypti* over the U.S. during winter months (December-March), except in southern portions of Florida and Texas that can sustain low-to-moderate potential mosquito abundance compared to summer (Fig. 2). Meteorological conditions are suitable for *Ae. aegypti* across all fifty cities during peak summer months (July-September), though the mosquito has not been observed in all cities. Highest potential mosquito abundances is simulated in the Southeast and south Texas where local cases of other *Aedes*-transmitted viruses have been reported previously, a result that is consistent with the suitability mapping studies noted above.

Introduction of the virus into local *Ae. aegypti* populations is more likely in cities with high volumes of people arriving from areas where transmission is ongoing (Fig. 1). This could include introduction by persons arriving in the U.S. by air, land (cross-border traffic by vehicles or walking) and sea (cruise ship passengers). Higher risk is thus implied for U.S.-Mexico border cities and neighboring areas, where population movement is high, and larger air traffic hubs such as Houston, Los Angeles or Miami. In Mexico, most reported Zika cases are in southern states, however cases have been reported further north in Sinaloa and Nuevo Leon by the Health Ministry of Mexico as of 22 February 2016. The potential for introduction in the U.S. may be highest during summer months when *Ae. aegypti* populations are abundant, particularly if Zika transmission increases in the northern Mexican states.

Multiple factors determine when and where arboviruses emerge; vector presence is only one factor that needs to be evaluated to assess the potential for disease emergence. Socioeconomic conditions that modulate human exposure to vectors play a role in population vulnerability to arboviruses^23^. Poverty is associated with lower air-conditioning use and less efficient cooling options^52^; such conditions can lead to open windows and doors and raise the probability of human-vector contact. Disparities in income have been associated with intermittent or no access to piped water in unregulated communities (colonias) in the U.S.-Mexico border region^65^; such conditions can necessitate water storage that in turn provides oviposition opportunities for *Ae. aegypti*. Intermittent garbage collection may also contribute to the provision of important oviposition sites along the U.S.-Mexico border. For example, discarded tires provide ideal habitat for *Ae. aegypti* due to their dark color, water holding capacity, and thermal insulation^66^. Additionally, the condition of housing infrastructure such as door and window screens may be lower in impoverished communities^53^. Areas in the southern parts of Texas demonstrated higher rates of poverty than some other regions under study (Fig. 3). Coupled with the higher levels of travel from areas where transmission is occurring and the history of *Ae. aegypti* –borne virus outbreaks, this suggests that southern Texas may be a vulnerable region for Zika transmission.

Even if Zika virus arrives in the United States, transmission can be mitigated and reduced through the use of vector control strategies. As part of our investigation we initially undertook a review of all vector control activities being conducted within these fifty cities. Due to the lack of standardization of reporting of *Ae. aegypti*-related activities in each jurisdiction we have not presented those results in this paper. It is clear, however, that vector control and public health interventions should consider tailoring their messages to the local communities. The provision of geographically referenced *Ae. aegypti* data to the public could assist in raising public awareness about the vector and motivate action when neighboring communities have *Ae. aegypti* activity^67^.

This study has numerous limitations related to the model simulations. We do not account for vector control practices in the simulations and thus abundance may be overestimated (as suggested during the off-peak months in Miami and Phoenix in the validation). There is incomplete understanding of how temperature may limit population dynamics at the geographic margins of *Ae. aegypti* survival^68^, where sensitivity to poorly-constrained meteorological thresholds may hamper model performance. For example, because knowledge of egg survival during winter in marginal areas is limited^68^, we artificially introduce eggs into the simulations each month, which keeps eggs from becoming extinct in cities that have seasonal *Ae. aegypti* populations, but likely causes egg availability/viability to be overestimated. Cryptic habitats that are not represented in models may enhance environmental suitability during winter; an overwintering *Ae. aegypti* population was recently found in Washington, D.C.^38^, suggesting that *Aedes* mosquitoes are adept at ovipositing in semi-concealed places that stay relatively warm year-round (e.g., subterranean habitats). Likewise, during the warm season it is likely that mosquitoes are adept at finding suitable microclimates in sheltered areas that aren’t explicitly accounted for in the models^29^, particularly in challenging environments such as desert cities where high temperatures and low humidity may otherwise inhibit mosquito survival. The influence of humidity in the simulations may not be adequately represented, which could be an important issue in arid regions where desiccation may substantially impact adult survival^62^. Little is known of the geographic distribution of container characteristics such as type, quantity and degree of shading, all factors that regulate water temperature and amount available for immature development. Additionally, the degree and frequency of manual filling of potential container habitats by humans is virtually unknown and may be over- or understated in the models. Intra-city climatic variability is not accounted for, which may be important in cites with sharp elevation gradients or heterogeneous urban land use patterns. Furthermore, the models do not allow for interaction between *Ae. aegypti* and other container dwelling invertebrates, including the competitor *Ae. albopictus*. This competitive interaction has been shown to determine the distribution of *Ae. aegypti* in Florida at scales of less than 5km^41^^,^ 69, and suggests the importance of understanding the local distribution of these mosquitoes in assessing risk. The competitive interaction may also affect the seasonality of *Ae. aegypti* due to increased competition late in the wet season^41^^,^ 70. The limitations noted here are the primary reason we refer to simulated *Ae. aegypti* abundance as potential abundance. Despite these limitations, our validation indicates that overall the Skeeter Buster and DyMSiM simulations, as well as their ensemble mean, resolve the seasonal cycle of *Ae. aegypti* abundance in Phoenix and Miami. Importantly, the simulations capture the months of peak abundance in both cities. We also find that Skeeter Buster and DyMSiM have similar seasonal cycles of abundance at most U.S. cities (Figs. S2 and S3).

Study limitations not related to the model simulations include incomplete information about travel from areas of ongoing Zika transmission. In particular, our air travel data set does not provide information on “thru-travelers” proceeding on to secondary and tertiary destinations from their U.S. port-of-entry. This likely leads to overestimated numbers for large international airports and underestimated numbers at smaller domestic airports. Additionally, though there are high numbers of travelers crossing the U.S.-Mexico border, many of the same individuals cross regularly, so actual unique individuals crossing is lower. As of February 2016 no Mexican cities along the border had local ZIKAV transmission, which suggests a low probability of introduction. Finally, our study region does not include non-contiguous U.S. states and territories where risk for transmission of *Aedes*-transmitted viruses is high: Hawaii, Puerto Rico, and areas in the South Pacific.

Though we do not explicitly address whether the potential abundance of *Ae. aegypti* may be lower or higher than normal in the forthcoming months of 2016 due to anomalous meteorological conditions (perhaps linked to the strong El Niño conditions occurring at the time of writing), it is noteworthy that a seasonal climate forecast initialized in February 2016 and valid for June-August 2016 suggests a 40-45% probability of above-normal temperatures over the entire contiguous U.S. for the upcoming summer of 2016^71^. This is compared to a 20-25% probability of below-normal temperatures, and a 35% probability of normal conditions. Therefore, it is possible that above-normal temperatures will lead to increased suitability for *Ae. aegypti* throughout much of the U.S. in summer 2016, though in some of the hottest regions of Texas, Arizona and California above-normal temperatures may lead to decreased suitability.

Despite the limitations, our analysis is a step towards simultaneously mapping the geographic and seasonal suitability of the vector mosquito *Ae. aegypti* in the contiguous United States. There is a need for enhanced, long-term, nationally-coordinated, local-level surveillance of both *Aedes* mosquitoes and *Aedes*-transmitted viruses, particularly in areas where simulations indicate *Ae. aegypti* populations may be high and coincide with more frequent travel between the U.S. and countries where Zika is circulating.

## Competing Interests

The authors declare no competing interests.

## Appendix

**Figure:**
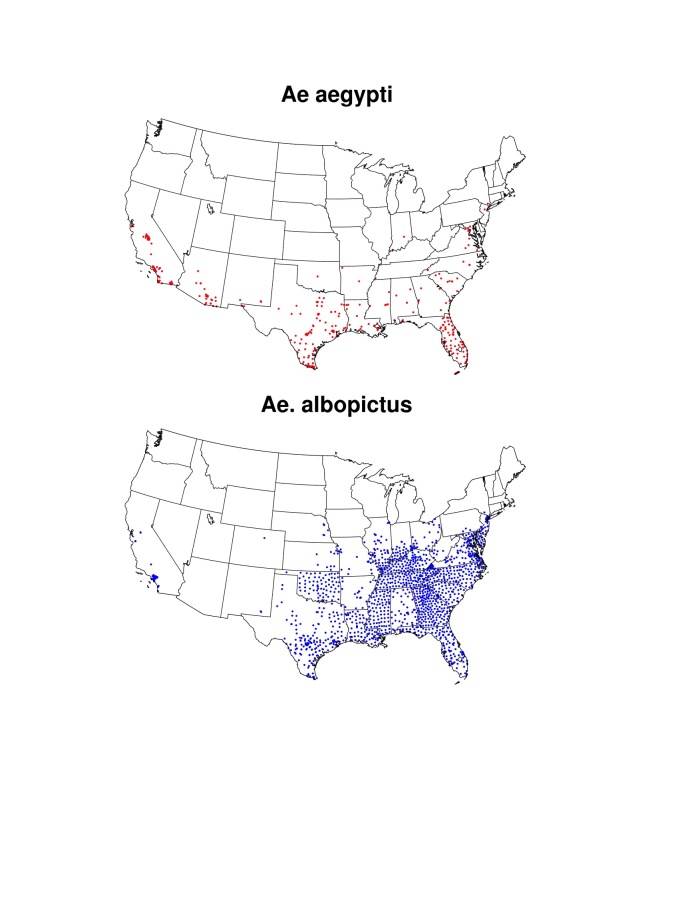
**Fig. S1.** Locations of know occurrences of *Ae. aegypti* and *Ae. albpictus* in the U.S. for 1960-2014 reproduced from Kraemer et al.^56^^,^ 57 ^,^ 58 and updated with collections in California for 2011-2015^59^ .

**Figure:**
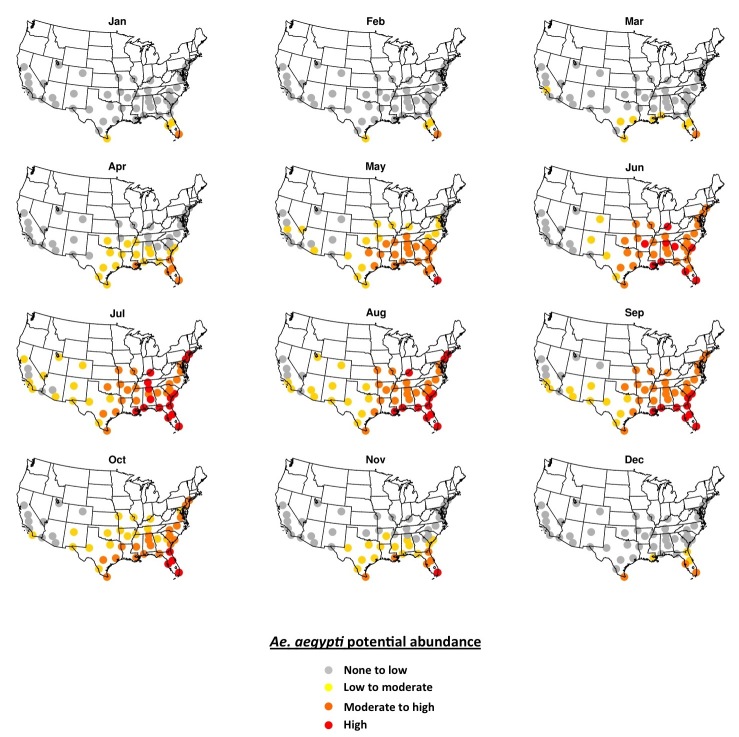
**Fig. S2.** The 2006-2015 Skeeter Buster mean monthly average *Ae. aegypti* potential abundance.

**Figure:**
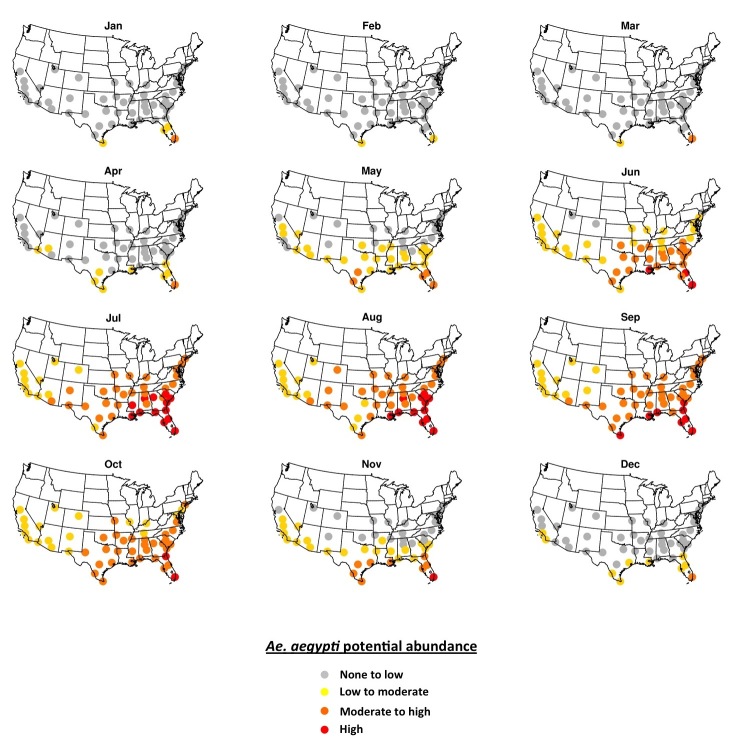
**Fig. S3.** The 2006-2015 DyMSiM mean monthly average *Ae. aegypti* potential abundance.
